# Intra Strain Variation of the Effects of Gram-Negative ESKAPE Pathogens on Intestinal Colonization, Host Viability, and Host Response in the Model Organism *Caenorhabditis elegans*

**DOI:** 10.3389/fmicb.2019.03113

**Published:** 2020-01-21

**Authors:** Euan Scott, Lindy Holden-Dye, Vincent O’Connor, Matthew E. Wand

**Affiliations:** ^1^School of Biological Sciences, University of Southampton, Southampton, United Kingdom; ^2^National Infection Service, Public Health England, Salisbury, United Kingdom

**Keywords:** *Caenorhabditis elegans*, *Klebsiella pneumoniae*, *Pseudomonas aeruginosa*, *Acinetobacter baumannii*, food aversion, pathogenicity, colonization, neuropeptide

## Abstract

In its native environment of rotting vegetation, the soil nematode *Caenorhabditis elegans* encounters a range of bacteria. This includes species from the ESKAPE group of pathogens that pose a clinical problem in acquired hospital infections. Here, we investigated three Gram-negative members of the ESKAPE group, *Pseudomonas aeruginosa*, *Klebsiella pneumoniae*, and *Acinetobacter baumannii*. Pathogenicity profiles as measured by time to kill adult *C. elegans* showed that *P. aeruginosa* was the most pathogenic, followed by *K. pneumoniae*, while *C. elegans* cultured on *A. baumannii* exhibited the same survival as those on the standard laboratory food source for *C. elegans*, *Escherichia coli* OP50. The pathogenicity was paralleled by a reduction in time that *C. elegans* resided on the bacterial lawn with the most pathogenic strains triggering an increase in the frequency of food-leaving. Previous reports indicate that gut colonization is a feature of pathogenicity, but we found that the most pathogenic strains were not associated with the highest level of colonization. Indeed, clearance of *P. aeruginosa* strains from the *C. elegans* gut was independent of bacterial pathogenicity. We show that this clearance is regulated by neuromodulation as *C. elegans* mutants in *unc-31* and *egl-3* have enhanced clearance of *P. aeruginosa*. Intriguingly this is also not linked to their pathogenicity. It is likely that there is a dynamic balance occurring in the *C. elegans* intestinal environment between maintaining a healthy, beneficial microbiota and removal of pathogenic bacteria.

## Introduction

The nematode worm *Caenorhabditis elegans* has been used to investigate several fundamental biological processes and has emerged as a useful model for bacterial infection (Brenner, 2009; Khan et al., 2018). *C. elegans* is a bacteriophagous free living nematode which resides in the soil and on rotting fruit or vegetation (Schulenburg and Félix, 2017). Ingestion of microorganisms in these habitats is reflected in the recent observation that environmental bacteria can colonize the worm gut generating a complex microbiota (Zhang et al., 2017). Pathogenic bacteria also exist in these environments and nematodes have evolved mechanisms to respond to bacterial pathogens (Cohen and Troemel, 2015). *C. elegans* can be infected by and reacts to bacterial pathogens including *Pseudomonas aeruginosa*, *Staphylococcus aureus*, *Salmonella enterica* serovar Typhimurium, and *Serratia marcescens*, as well as several non-human bacterial pathogens (Tan et al., 1999a; Aballay et al., 2000, 2003; Garsin et al., 2001; Kurz et al., 2003). Bacterial pathogenicity in *C. elegans* can be investigated using several approaches including fast and slow killing (Darby et al., 1999; Mahajan-Miklos et al., 1999; Tan et al., 1999a). There is also increasing evidence that measures of *C. elegans* behavior in response to bacteria can be a useful surrogate of the underlying pathogenicity that ultimately progresses to death (Meisel and Kim, 2014). *C. elegans* can remain on bacterial lawns for extended periods of time which is known as dwelling behavior (Ben Arous et al., 2009). On lawns that contain potentially pathogenic bacteria or other aversive perturbations, *C. elegans* exhibit increased motility, food leaving events, and overall increased time off the lawn (Melo and Ruvkun, 2012). This is described as enhancement of food leaving or food aversion behavior (Melo and Ruvkun, 2012). These behaviors are also similar to other learnt behaviors of *C. elegans* in relation to aversive or repellent odorants or other chemicals (Meisel and Kim, 2014). This indicates that neuromodulatory pathways in *C. elegans* can be activated as part of the host response to distinct bacterial pathogens to enable the worms survival (Mills et al., 2012).

In this study, *C. elegans* was utilized to compare a selection of Gram-negative opportunistic pathogens. We provide insight into the relative pathogenicity of five strains each of *Klebsiella pneumoniae*, *Acinetobacter baumannii*, and *P. aeruginosa* (members of the ESKAPE group of pathogens). These pathogens are becoming more problematic in a clinical environment, especially in infections with immunocompromised individuals (Brenner, 1974) and therefore understanding their virulence is of fundamental importance. The well-defined pathogenic strain in *C. elegans P. aeruginosa* PA14 was used as a known comparison. We relate pathogen induced killing, nematode food aversion, and bacterial gut colonization. Results shown in this study reinforce the emergent view for complex host–pathogen interactions by implying several interacting determinants are controlling bacterial pathogenicity in *C. elegans*. Furthermore, through analysis of specific mutants that are defective in neuromodulation, we show that the nervous system is actively engaged in regulating the gut microbiome.

## Materials and Methods

### *C. elegans* and Bacterial Strain Maintenance

*Caenorhabditis elegans* strains used in this study were Bristol N2, DA509 *unc-31 (e928*) IV, XA3741 *egl-3* (*ok979*). All strains were maintained on 5 cm Nematode growth media (NGM) plates with *Escherichia coli* OP50 as a food source according to standard methods (Brenner, 1974). Infection with *Galleria mellonella* was performed according to previous protocols (Wand et al., 2012). The bacterial strains used in this study are clinical strains with a variety of drug resistance markers that have previously been studied and are listed in Table 1 (Wand et al., 2012, 2013; Benthall et al., 2015). The experiments used numerical coding (P1-5, K1-5, and A1-5) to define the different bacterial strains and were therefore performed blind as to the identity of the bacteria with the code only being revealed at the end of the experiments conducted in this paper; the original strain names are used throughout the manuscript. Bacterial MLST typing was based on the Pasteur MLST typing. All strains were grown in LB broth with aeration or on LB agar plates at 37°C from glycerol stocks kept at −80°C unless otherwise stated.

**TABLE 1:** List of strains used in this study.

**Species**	**Strain**	**Sequence type (ST)**	**Relevant antibiotic resistance genes**
*Klebsiella pneumoniae*	NCTC 9633	3	*blaSHV-1*
	NCTC 13438	258	*aadA2, aph(3’)-Ia, blaKPC-3, blaSHV-11, blaOXA-9, gyrA* S83I, *parC* S80I, *mphA, catA1, dfrA12*
	MGH 78578	38	*aadB, aph(3’)-Ic, aadA1, aac(6’)-Ib, blaSHV-11, blaSHV-12, blaTEM-1B, blaOXA-9, gyrA* S83Y, *cmlA1, catA1, tetD*
	NCTC 13439	54	*aacA4, aac(6’)Ib-cr, blaSHV-12, blaVIM-1, QnrS1*
	NCTC 13368	489	*aadB, blaSHV-18, blaOKP-B-7, blaOXA-2*
*Acinetobacter baumannii*	UKA15	25	*aac(3)-IIa, blaOXA-23, blaOXA-64, blaADC-25, gyrA* S81L, *parC* S84L, *tetB*
	UKA2	2	*aac(3)-Ia, blaOXA-83, blaTEM-1D, blaADC-25, gyrA* S81L, *parC* S84L, *tetB*
	AYE	1	*aadB, aac(3)-Ia, aph(3’)-Ic, blaADC-25, blaOXA-10, blaVEB-1, blaOXA-69, gyrA* S81L, *parC* S84L, *catA1, cmlA1, ARR-2, tetA, tetG, dfrA1, dfrA10*
	ATCC 17978	ND	*blaADC-25, blaOXA-180*
	W1	1	*aacA4, blaOXA-69, blaADC-25, aac(6’)Ib-cr, gyrA* S81L, *parC* S84L
*Pseudomonas aeruginosa*	PAO1	549	*aph(3’)-IIb, blaOXA-50, blaPDC-1, catB7*
	GH56	395	*aph(3’)-IIb, blaOXA-50, blaPDC, catB7*
	GH12	235	*aph(3’)-IIb, aph(3’)-VIa, aadA6, aac(6’)-I1, aac(6’)-31, blaOXA-2, blaOXA-488, blaPDC, blaVEB-1, catB7, gyrA* T83I, *parC* S87L, *tetA, tetG*
	GH97	111	*aph(3’)-IIb, blaOXA-50, blaPDC, catB7*
	NCTC 13359	252	*aph(3’)-IIb, blaOXA-50, blaPDC, catB7*
	PA14	253	*aph(3’)-IIb, blaOXA-488, blaPDC-34, catB*

*Sequence type (ST) is a measure of the relatedness of each isolate utilizing the variation in sequence of specific genes. ND = not defined.*

### Preparation of Assay Plates

Bacterial cultures were prepared from cultures grown overnight at 37°C in 5 mL of LB broth. The following day a 100 μL sample of the overnight culture was sub-cultured into 10 mL of LB at 37°C and grown until an OD^600^ of 0.8 was reached; 50 μL of this fresh culture was pipetted onto the center of 5 cm NGM plates and left to grow overnight at 20°C to form a lawn for *C. elegans* behavioral experiments.

### *C. elegans* Killing Assay

15-20 L4 plus 1 *C. elegans* were transferred onto plates prepared as described above. Worms were observed each day and recorded as dead when they did not respond to being touched with a platinum wire. Animals that had crawled off the side of the plate were censured and removed from the assay. Individual worm populations were transferred to a new NGM plate with a fresh lawn of the same bacteria every other day to ensure the original adult population was the only one being observed and were devoid of any progeny produced during the experiment. The life span of *C. elegans* cultivated on the distinct bacterial strains was measured to assess the relative killing time. The data were plotted in a Kaplan Meier plot with the mean and 95% killing time being measured.

### *C. elegans* Behavioral Assays

*Caenorhabditis elegans* populations were developmentally synchronized by picking L4 larval stage defined by the presence of a developing vulva. L4 worms were then maintained overnight on OP50 to become L4 plus 1 (young adults). At the start of the assay, seven young L4 plus 1 *C. elegans* were transferred onto the middle of the bacterial lawn and observed at 2, 6, 24, and 48 h after this initial transfer (Supplementary Figure S1). Food aversion was measured at these time points by the proportion of the original seven worms that were off the bacterial lawn. For egg laying and progeny production, previous published methods were utilized (Scott et al., 2017).

### Determination of *C. elegans* Bacterial Colonization

Determining the number of bacterial cells present in the *C. elegans* intestine was based on previous protocols (Begun et al., 2007; Kawli and Tan, 2008; Portal-Celhay et al., 2012). Following an indicated time of exposure to the bacteria, approximately 10 worms were paralyzed by being picked into a 5 μL drop of 25 mM levamisole in M9 buffer on an unseeded 5 cm NGM plate. These paralyzed worms with inhibited pharyngeal pumping were washed three times with 1 mM sodium azide. The worms were then further washed twice with 5 μL drops of M9. For homogenization, *C. elegans* were picked into an Eppendorf containing sterile PBS with 1% Triton X-100 and lyzed using a glass pestle. The exact number of worms that lyzed was analyzed by visual inspection. The worm lysates were then serially diluted in PBS and plated on LB agar. Following overnight growth at 37°C, the number of colony forming units (CFUs) per animal was calculated. For experiments where *P. aeruginosa* colonization was measured after being transferred to OP50, the diluted worm lysates were plated on cetrimide agar for specific selection of *P. aeruginosa.* The pharyngeal pumping rate of *C. elegans* was measured by observing worms under 60x magnification while the worms were feeding on bacterial lawns. One contraction and relaxation of the grinder portion of the terminal bulb was considered one pharyngeal pumping event. This was measured continuously for 60 s.

### Measure of Clearance of Bacteria From the *C. elegans* Intestine

Animals were fed on a lawn of fresh *P. aeruginosa* for 24 h and then analyzed for bacterial colonization as described above to measure the number of bacteria colonizing *C. elegans*. In addition, *C. elegans* was also exposed to *P. aeruginosa* for 24 h and then transferred to a lawn of OP50 for an additional 24 h. Animals were analyzed for *P. aeruginosa* CFUs as above by being plated on cetrimide agar. To calculate the efficacy with which *P. aeruginosa* cells are removed from *C. elegans*, the fold clearance was calculated by dividing the number of *P. aeruginosa* CFUs at 24 h by the number of *P. aeruginosa* measured 24 h post-OP50 transfer.

### Statistical Analysis

All statistical analysis was done in Graphpad Prism and the individual tests used as indicated in figure legends). ^∗^*P* < 0.05, ^∗∗^*P* < 0.01, ^∗∗∗^*P* < 0.001, ^****^P < 0.0001.

## Results

**FIGURE 1:**
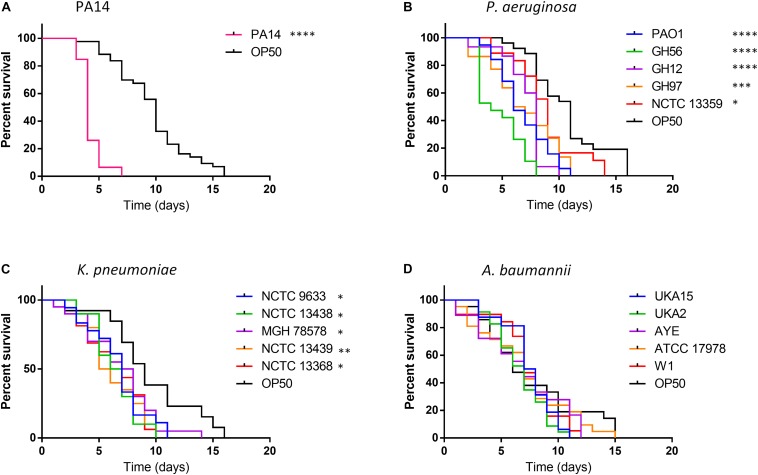
Survival of *C. elegans* exposed to strains of *P. aeruginosa*, *K. pneumoniae*, and *A. baumannii* relative to *E. coli* OP50. *C. elegans* survival after challenge with *P. aeruginosa* PA14 **(A)**, other *P. aeruginosa* strains **(B)**, *K. pneumoniae* **(C)** and *A. baumannii* **(D)** was recorded every day until 100% mortality was observed in the *E. coli* OP50 control. A log-rank (Mantel–Cox) test was used to compare the mortality of all strains relative to OP50 and significance is indicated. ^∗^*P* < 0.05, ^∗∗^*P* < 0.01, ^∗∗∗^*P* < 0.001, ^****^P < 0.0001.

The initial aim was to identify the pathogenicity of the 15 clinical strains of *P. aeruginosa*, *K. pneumoniae*, and *A. baumannii* relative to *P. aeruginosa* strain PA14. These strains were selected because they had previously shown a range of lethality in another invertebrate model *G. mellonella* (Supplementary Figure S2) (Wand et al., 2012, 2013; Benthall et al., 2015). For *P. aeruginosa*, which is naturally highly virulent in *Galleria* with an infectious dose of less than 100 CFUs, only one strain (GH97) showed reduced virulence in *Galleria*. To understand which strains were more pathogenic in *C. elegans*, worms were exposed to each bacterial strain at 20°C until death was observed (Figure 1). These killing experiments were designed to mimic the conditions on which a standard lifespan of *C. elegans* would be measured with the animals feeding on the laboratory standard food source of *E. coli* OP50 alone.

Exposure to OP50 led to total mortality after 16 days, conversely killing of *C. elegans* with the hypervirulent *P. aeruginosa* PA14 was more rapid with all worms’ dead after 7 days exposure (Figure 1A). For other *P. aeruginosa* strains tested killing was enhanced relative to OP50 with total mortality occurring between 8–11 days for all strains except NCTC 13359 (14 days) with strain GH56 showing a *C. elegans* mortality profile similar to PA14 (Figure 1B). This strain showed enhanced virulence relative to the other *P. aeruginosa* strains (Supplementary Table S1). All *K. pneumoniae* strains tested also showed significantly enhanced killing relative to OP50 with total *C. elegans* mortality occurring 10–14 days for all strains after initial challenge (Figure 1C). For *A. baumannii*, there appeared to be no significant time difference in mortality between any of the strains and OP50 (Figure 1D). Unlike *P. aeruginosa*, *K. pneumoniae* and *A. baumannii* showed little strain to strain variation in virulence in *C. elegans* (Supplementary Table S1). Due to the variation in virulence with *P. aeruginosa* strains, the whole genome sequences were analyzed for the presence of potential virulence factors. GacA and ToxA have been shown to be important virulence factors for slow killing of *C. elegans* by *P. aeruginosa* (Tan et al., 1999b). Analysis of the amino acid sequences for these proteins showed slight sequence variation but this and the presence/absence of other specific virulence factors previously described (Gallagher and Manoil, 2001; Feinbaum et al., 2012) cannot readily explain the enhanced virulence of *P. aeruginosa* strains PA14 and GH56 (Supplementary Table S2).

### *C. elegans* Food Aversion Behavior to the Range of Bacterial Pathogens

**FIGURE 2:**
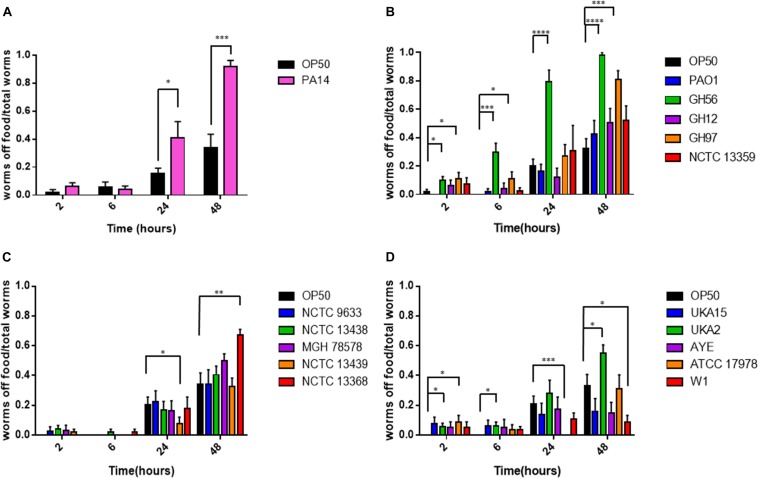
Food aversion for different bacterial pathogens relative to *E. coli* OP50. The proportion of worms off food for *P. aeruginosa* PA14 **(A)**, other *P. aeruginosa* strains **(B)**, *K. pneumoniae* **(C)**, and *A. baumannii* **(D)** was measured at various time-points after exposure to the bacterial lawn as shown. These experiments were performed in at least triplicate for all strains. Error bars represent ± SEM. Analysis is by one-way ANOVA with Tukey’s multiple comparison. Significance relative to OP50 at the relevant time-point is shown. ^∗^*P* < 0.05, ^∗∗^*P* < 0.01, ^∗∗∗^*P* < 0.001, ^****^P < 0.0001.

**FIGURE 3:**
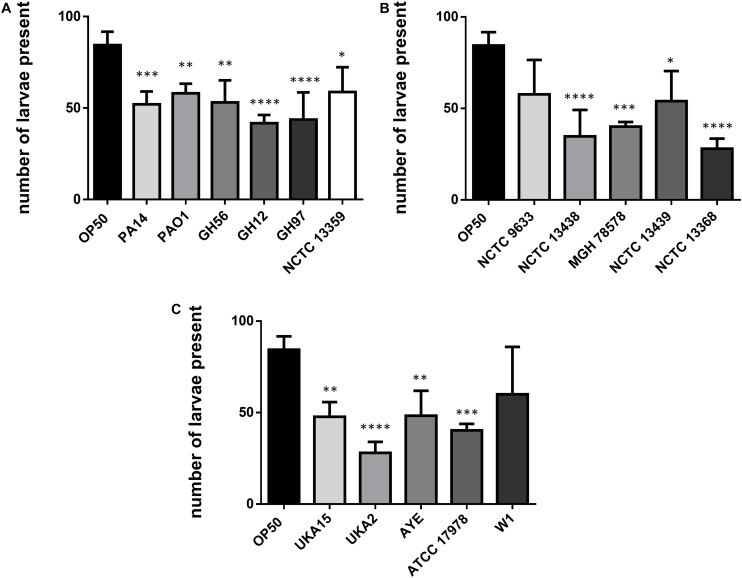
Number of *C. elegans* progeny after 48 h exposure to bacterial pathogens. Results are shown for *P. aeruginosa* **(A)**, *K. pneumoniae* **(B)**, and *A. baumannii* **(C)** strains and were compared to *E. coli* OP50. These experiments were performed in at least triplicate for all strains. Error bars represent ± SEM. Analysis is by one-way ANOVA with Tukey’s multiple comparison. Significance relative to OP50 is shown. ^∗^*P* < 0.05, ^∗∗^*P* < 0.01, ^∗∗∗^*P* < 0.001, ^****^P < 0.0001.

*Caenorhabditis elegans* has been shown to exhibit food aversion when exposed to various pathogenic bacteria. Therefore, we sought to understand whether measurements of food aversion are indicative of pathogenicity and worm killing. The number of worms off the bacterial lawn was measured at given time-points (2, 6, 24, and 48 h after initial exposure). Exposing adult wild-type *C. elegans* to lawns of OP50 showed that as time increased, the number of worms existing off the food source increased. In comparison, there was a greater tendency for *C. elegans* to remain off the food source if that food source was *P. aeruginosa* PA14 (Figure 2A). This was also true for two other *P. aeruginosa* strains (GH56 and GH97) (Figure 2B). For *K. pneumoniae* (Figure 2C) and *A. baumannii* (Figure 2D), there was little difference in enhanced food aversion with only challenge with *K. pneumoniae* strain NCTC 13368 and *A. baumannii* strain UKA2 leading to an increased number of worms off the food at the 48 h time-point. Indeed, certain *A. baumannii* strains as a food source appeared to show reduced food aversion relative to OP50 at the later time points (strains ATCC 17978 and W1). This could be caused by visual depletion of the OP50 lawn relative to the bacterial lawns from the ESKAPE pathogens. Visual inspection of the lawns from all *A. baumannii*, *K. pneumoniae*, and *P. aeruginosa* strains did show that there was no noticeable depletion of the bacterial lawns throughout the 48 h assay with the lawns of some strains, specifically certain *K. pneumoniae* strains, becoming thicker. This is reflected in their mucoid phenotype which is linked to the presence of the *rmpA* gene (Hsu et al., 2011). The screen for food aversion suggested that exposure of *C. elegans* to *K. pneumoniae* NCTC 13368, *A. baumannii* UKA2, *P. aeruginosa* GH56, and *P. aeruginosa* GH97 generated an aversive response as measured by an increased propensity to be off the bacterial lawn. Those strains that resulted in enhanced levels of food aversion were also pathogenic indicating that enhanced food aversion can be a predictor of bacterial pathogenicity in *C. elegans*. Egg laying and hatching, and the ability to develop larval progeny are measures of *C. elegans* health. Worms which are maintained in environments containing harmful chemicals or varying quality of food might be expected, given their reduced time present on food, to produce fewer eggs. When egg laying and progeny production were investigated, there was found to be no significant difference between the number of eggs present on the bacterial lawn at 24 h between any of the strains and OP50 (Supplementary Figure S3). However, the number of progeny at 48 h was significantly reduced for all strains relative to OP50 except for strains *K. pneumoniae* NCTC 9633 and *A. baumannii* W1 (Figure 3).

### Colonization Levels of Bacteria in *C. elegans*

**FIGURE 4:**
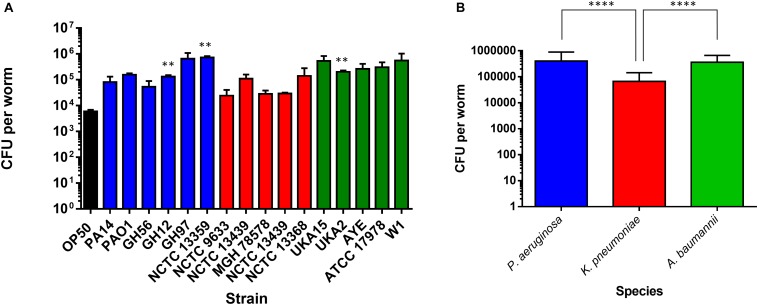
Colonization of the *C. elegans* intestine by bacterial pathogens. The ability of strains of *P. aeruginosa*, *K. pneumoniae*, and *A. baumannii* to colonize the *C. elegans* intestine was measured after 48 h exposure **(A)** and compared to *E. coli* OP50. These experiments were performed in at least triplicate for all strains. Error bars represent ± SEM. Analysis is by one-way ANOVA with Tukey’s multiple comparison. Significance relative to OP50 is shown. Comparison of mean CFU per species of bacteria in colonization of *C. elegans* after 48 h exposure **(B)**. Error bars represent ± SEM. Analysis is by student’s unpaired *t*-test and significance relative to the other bacterial species is shown. ^∗^*P* < 0.05, ^∗∗^*P* < 0.01, ^∗∗∗^*P* < 0.001, ^****^P < 0.0001.

To investigate possible determinants for the spectrum of virulence exhibited in the range of strains we examined the ability of all strains to colonize the gut of *C. elegans* after ingestion. Worms were exposed to individual bacterial strains for 48 h (the last time-point in the food aversion assay) after which the *C. elegans* were homogenized and the number of CFUs per worm calculated (Figure 4A). There were relatively few strains which showed a significant change in the bacterial number isolated after 48 h relative to OP50. Statistical analysis revealed that there was no significant variation between the colonization levels of any two bacterial strains when compared intra-species. However, when the average colonization levels for each species of bacteria was compared with the others, it was shown that *K. pneumoniae* had significantly reduced colonization numbers relative to *P. aeruginosa* and *A. baumannii* (Figure 4B). To understand whether colonization levels were affected by the rate of *C. elegans* ingestion of bacteria, the pharyngeal pumping rate was measured at 48 h relative to OP50 while the worms were feeding. For all strains tested, the pumping rate per minute was approximately 240. There was no significant change between OP50 and the other strains tested with the exception of PA14 exposed animals which had reduced pharyngeal pumping with a rate of 223 pumps per minute.

Previous studies investigating PA14 had proposed that colonization was an important determinant of pathogenicity. To extend our comparative observations which suggest that this is not the case, we considered the time taken to cause death in 75% of worms. When *P. aeruginosa* strains were compared with OP50, there appeared to be no significant difference between colonization levels at 48 h and the longer time-points, and no significant differences between the *P. aeruginosa* strains and OP50 (Supplementary Figure S4).

**FIGURE 5:**
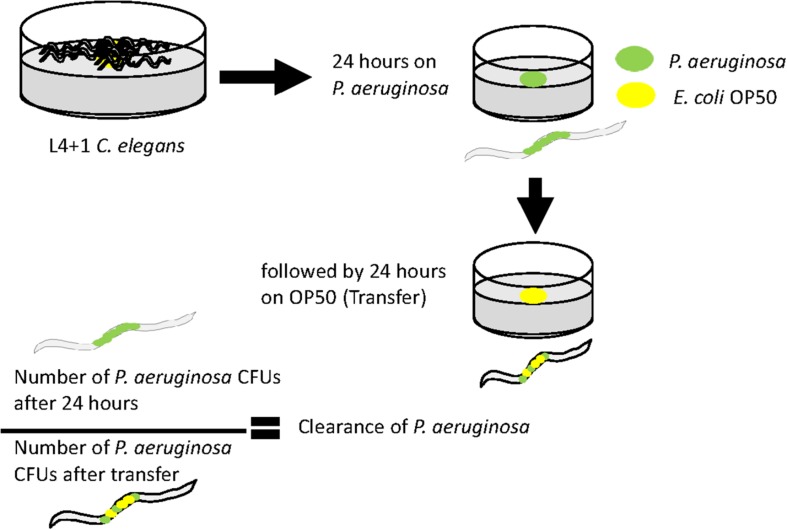
Methods for measuring clearance of *P. aeruginosa* from the *C. elegans* intestine after transfer to lawns of *E. coli* OP50. Age-synchronized 1 day old adult hermaphrodites were grown on a lawn of each *P. aeruginosa* strain before transfer to a lawn of *E. coli* OP50 for a further 24 h. Worms were analyzed for *P. aeruginosa* content immediately before and after transfer to *E. coli* OP50. The ratio of the CFU’s at each of these time points is an indicator of the clearance of *P. aeruginosa* from the intestine during exposure to *E. coli*.

**FIGURE 6:**
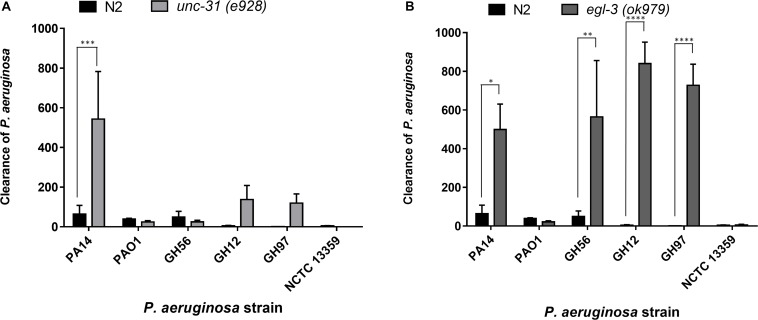
Clearance of *P. aeruginosa* strains from the *C. elegans* intestine is highly dependent on intact neuromodulation. The clearance of different strains of *P. aeruginosa* from the *C. elegans* intestine in *unc-31 (e928)* **(A)** and *egl-3 (ok979)* **(B)** mutant worms was measured using methods outlined in Figure 5. These experiments were performed in at least triplicate for all strains. Error bars represent ± SEM. Analysis is by one-way ANOVA with Sidaks multiple comparison. Significance relative to N2 worms is shown. ^∗^*P* < 0.05, ^∗∗^*P* < 0.01, ^∗∗∗^*P* < 0.001, ^****^P < 0.0001.

Since the levels of colonization at 48 h did not appear to correlate with pathogenicity, we proceeded to examine the ability of the colonizing bacteria to be cleared from the *C. elegans* intestine once the worms were transferred to an *E. coli* OP50 food source (Figure 5). We focused on *P. aeruginosa* strains since these exhibited the greatest variation in killing (Figure 1B) and for comparison with PA14. Results showed that after exposure to individual *P. aeruginosa* strains for 24 h strains PA14, PAO1, and GH56 were cleared more rapidly following additional 24 h exposure to OP50 (Figure 6). Apart from PAO1 having enhanced clearance relative to strains GH12, GH97, and NCTC 13359, there was no other significant difference in the clearance of any *P. aeruginosa* strains relative to another. We did notice increased variation in clearance rates in PA14 and GH56 which may account for this lack of significance in clearance rates, although these results do indicate that *C. elegans* is able to clear more pathogenic strains (PA14, GH56) with the same efficiency as those which are less pathogenic (NCTC 13359).

Previous research has highlighted the importance of the neural network in pathogen avoidance (Cao et al., 2017). It has also indicated that *C. elegans* mutants with deficiencies in neuronal signaling show enhanced clearance rates of pathogenic bacteria after transfer to a non-pathogenic food source (Kawli and Tan, 2008). These previous experiments concluded a critical role for neuropeptide mediated modulation of neural signaling in the processes that control gut clearance. This was based on deficiencies in mutants that play a role in vesicle mediated exocytosis that initiates such neural signaling. Here we extended this analysis and investigated clearance from distinct mutants that have identified roles in peptide function but have not been investigated in the context of gut clearance. Clearance experiments using the mutant *unc-31(e928)* nematode, which is deficient in several aspects of neuronal function including neuropeptide signaling, revealed that PA14 is cleared more effectively in mutant *unc-31(e928)* worms than N2 worms (Figure 6A), but that clearance for other *P. aeruginosa* strains appeared to not be significantly enhanced. This suggested that the comparative approach provided an intersectional insight to further probe the relationship between neuromodulation and a generic role in bacterial pathogenicity. We also examined clearance rates in *egl-3(ok979)* worms, which are deficient in neuropeptide processing and lacks > 100 important neuropeptide transmitters including the previously implicated insulin related peptide INS-7 (Kawli and Tan, 2008). There was enhanced clearance of four of the *P. aeruginosa* strains (PA14, GH56, GH12, GH97) in these mutants relative to N2 worms but not for two others (PAO1 and NCTC 13359) (Figure 6B). This observation suggests a broader impact of Egl-3 and peptide processing (i.e., the ability to generate bioactive peptides) over determinants like Unc-31 and Unc-64 (Kawli and Tan, 2008) that mediate peptide secretion.

## Discussion

Robust models of bacterial pathogenesis are required to address the challenges presented by antimicrobial resistance. *C. elegans* has emerged as a promising model for investigating bacterial infection and virulence (Marsh and May, 2012). However, previous studies have only investigated a very limited number of strains. In this study, we systemically screened several *K. pneumoniae*, *A. baumannii*, and *P. aeruginosa* isolates to determine their pathogenicity against *C. elegans*. We related this to their aversive influence on *C. elegans* behavior and provide insight into the differential response of the nematode to infections with varying pathogenicity.

The ability of *C. elegans* to regulate lawn occupancy is governed by food abundance and quality (Shtonda and Avery, 2006; Milward et al., 2011). *C. elegans* will move off lawns of more pathogenic bacteria (Anderson and McMullan, 2018) and results here show that those strains which are more pathogenic (*P. aeruginosa* GH56 and PA14), as measured by time to kill *C. elegans*, also resulted in an increased food aversion. This aversion is not linked to food depletion since visual inspection of the lawns indicated that lawns from all ESKAPE bacteria were intact and this is likely reflected in the different nutrient requirements for these bacteria relative to OP50. Behavior linked to food aversion is governed by components of the sensory neurons such as *tax-2* and *tax-4* (Milward et al., 2011). Experiments on food aversion using mutants in these genes suggest that this aversion is an exteroceptive response to bacteria after ingestion (Milward et al., 2011). The residence time of *C. elegans* on bacterial lawns is also regulated by population density and the presence of larvae; as the population increases, worms are more likely to leave the bacterial lawn (Milward et al., 2011; Scott et al., 2017). It is important to note that all the pathogenic strains of bacteria tested here reduced the fecundity of *C. elegans* resulting in lower numbers of larvae appearing over 24 h compared to worms cultured on *E. coli* OP50. Therefore, the increased food aversion observed with the pathogenic strains is not an indirect consequence of an effect on population density. The number of *C. elegans* progeny produced is not directly related to the number of eggs on the bacterial lawn indicating that while the presence of these pathogenic ESKAPE pathogens has no significant effect on egg production, it does imply an effect on egg hatching.

Bacteria can avoid lysis by the grinder section of the *C. elegans* pharynx (Avery and Shtonda, 2003) and colonize the post pharyngeal intestine in *C. elegans*. The ability of different bacteria to colonize the *C. elegans* intestine is an important determinant of virulence and pathogenicity (Kawli and Tan, 2008). In addition, previous studies using *P. aeruginosa* have implicated the nematode gut colonization in driving facets of the pathogenic responses (Schulenburg and Ewbank, 2004). However, our results show that all strains from the same species colonized *C. elegans* to a similar degree which was not dependent upon their relative pathogenicity. Longer term colonization with *P. aeruginosa* did also not appear to differentiate between strains with different pathogenicity profiles. This observation that colonization is not the primary determinant of pathogenicity is consistent with a previous study which showed that slow killing of *C. elegans* by strains of *Burkholderia pseudomallei* occurs in the absence of gut colonization (Ooi et al., 2012). In our analysis, colonization appeared to be species rather than strain dependent with higher numbers of bacteria isolated from the *C. elegans* gut colonized with *A. baumannii* or *P. aeruginosa* than *K. pneumoniae* strains. Therefore, *C. elegans* gut conditions may be more amenable to *A. baumannii* and *P. aeruginosa* colonization or these bacteria are better equipped to bypass the pharyngeal system of *C. elegans* (Avery and Shtonda, 2003).

Bacterial colonization of the intestine can be reversed by the response of the nematode to the infection. We found that wild-type (N2) *C. elegans* showed variation in the clearance levels for different *P. aeruginosa* strains but that overall bacterial pathogenicity was not a simple predictor of the clearance response. Thus, this more extensive comparison of pathogenicity for a range of bacterial isolates reveals that the determinants which drive interactions between the nematode host and the bacterial pathogen are more complex than previously appreciated. The enhanced clearance of specific *P. aeruginosa* strains from the *C. elegans* intestine using *egl-3* (Kass et al., 2001) and *unc-31* mutants (Avery et al., 1993), which are disrupted in aspects of neuropeptide maturation and release, respectively, showed the importance of these proteins in post bacterial colonization clearance. Indeed, there is evidence that *unc-31* mutants are more resistant to infection with *P. aeruginosa* PA14 due to the expression of increased levels of immune effectors (Evans et al., 2008). However, the absence of these genes has no effect on the clearance of *P. aeruginosa* strains PAO1 and NCTC 13359. This suggests that while having a fully functioning range of neuropeptides reduces the number of bacteria that are cleared from *C. elegans*, there are other unknown aspects which are equally important in bacterial clearance (Montalvo-Katz et al., 2013).

The existence of a complex *C. elegans* gut microbiota which aids in protection against infection by pathogens has been reported (Kissoyan et al., 2019). The *C. elegans* gut microbiota was found to include *Pseudomonas* species and Enterobacteriaceae including *Klebsiella* species (Dirksen et al., 2016). It was observed that the four strains which are more highly cleared by *egl-3* mutants included *P. aeruginosa* PA14 and GH56, the most pathogenic strains based on previous killing experiments, suggesting that this enhanced clearance in the mutant may be more selective toward more harmful strains. In addition, the more general effect of Egl-3 which is essential in proteolytic processing that converts pro-proteins to their bioactive peptides indicates that this biological function, rather than peptide secretion, is more important in defining the landscape of the bacterial host interaction. In this regard, it should be noted that Egl-3 although primarily associated in the processing of prohormones is also responsible for generating processed peptides that encode extracellular adhesion or bactericidal activities that could clearly impact host–pathogen interactions (Thacker and Rose, 2000; Dierking et al., 2016). This further suggests that colonizing bacteria elicit a differential response from the worm depending on the nature of the interaction occurring in the intestinal tissue and may indicate that specific *Klebsiella* and *Pseudomonas* strains are treated as commensals rather than pathogenic. Studies on the natural microbiome of *C. elegans* have shown that its physiology is affected differently by isolates from the same genera. This suggests that genetic differences at the strain level are important in pathogenicity (Samuel et al., 2016). Our results are consistent with this. While there was a clear trend suggesting that *P. aeruginosa* strains were more pathogenic than *K. pneumoniae* which in turn was more pathogenic than *A. baumannii*, there was strain to strain variation not just in terms of ability to kill *C. elegans* but also in terms of food aversion and clearance. All *A. baumannii* strains had similar virulence and food aversion profiles compared to *E. coli* OP50, which was observed in previous studies (Vallejo et al., 2015), but appeared to colonize in greater numbers.

This study has shown evidence of a multi-faceted host response due to bacterial colonization and that the nematode responses are driven by the properties of the bacterial strain or species. The evidence presented here indicates that neuropeptides and/or neuromodulation are important in regulating this balance but are not the only factor. It is likely that there is a dynamic balance occurring in the *C. elegans* intestinal environment between maintaining a healthy beneficial microbiota and removal of pathogenic bacteria. This would be especially true in the natural environment of *C. elegans* when animals are constantly moving between different bacterial food sources. The comparative approach we have used here highlights the rich biology of bacterial host interactions in *C. elegans* (Zhang et al., 2017) and should inform on the use of model organisms utilizing both environmental and clinical relevant strains for studying bacterial pathogenicity in *C. elegans*.

## Data Availability Statement

All datasets generated for this study are included in the article/Supplementary Material.

## Author Contributions

ES and MW performed *in vitro* experiments. All the authors analyzed the data, designed the study, wrote the manuscript, read, and approved the final version of the manuscript.

## Conflict of Interest

The authors declare that the research was conducted in the absence of any commercial or financial relationships that could be construed as a potential conflict of interest.
