# Supplementary material for: Intra Strain Variation of the Effects of Gram-Negative ESKAPE Pathogens on Intestinal Colonization, Host Viability, and Host Response in the Model Organism Caenorhabditis elegans
Source: Front Microbiol. 2020 Jan 21;10:3113. doi: 10.3389/fmicb.2019.03113 (PMC6985274; doi:10.3389/fmicb.2019.03113)
Supplement: Supplementary file 1 [file Data_Sheet_1.docx]

**Supplementary Tables and Figures.**

Supplementary Figure S1

NGM plate seeded with 50µl of OD₆₀₀= 0.8 culture of bacteria and left to grow into a lawn

7x young adult *C. elegans* transferred onto plate

Worms off food

Worms on food

Proportion of worms off food measured at 2, 6, 24 and 48 hours after transfer to plate

**Figure S1 -** Setup of a *C. elegans* food aversion assay on NGM agar.

Supplementary Figure S2

*P. aeruginosa*

*K. pneumoniae*

*A. baumannii*

Figure S2 – Virulence in *G. mellonella* of strains of *P. aeruginosa*, *K. pneumoniae* and *A. baumannii*. *Galleria* were injected with *P. aeruginosa* (Input 1 x 10^2^ CFU), *K. pneumoniae* (1 x10^5^ CFU) and *A. baumannii* (1 x 10^6^ CFU) and the number of larvae alive scored periodically every 24 hrs for 120 hrs. Results are shown for individual strain infection using 30 *Galleria* per strain*.*

Supplementary Figure S3

A

B

C

Figure S3 –Egg Production after 24 hrs exposure to *P. aeruginosa* (A), *K.* *pneumoniae* (B) and *A. baumannii* (C) strains. Experiments were performed in triplicate for all strains. Error bars represent ±SEM. Analysis is by One-Way ANOVA with Tukey’s multiple comparison.

Supplementary Figure S4

Figure S4 – Long term colonisation of *P. aeruginosa* strains in *C. elegans*. The number of bacteria colonisation individual *C. elegans* worms was measured at 2 days and the timepoint where 75% of worms had died (e.g. for OP50 this was 13 days and for PA14 this was 5 days). All results were performed in triplicate. Error bars represent ±SEM. Analysis is by One-Way ANOVA with Tukey’s multiple comparison.

Supplementary Table 1

| *P. aeruginosa* | PAO1 | GH56 | GH12 | GH97 | NCTC 13359 |
| --- | --- | --- | --- | --- | --- |
| PAO1 |  | 0.0102 | 0.8226 | 0.5307 | 0.0304 |
| GH56 | 0.0102 |  | 0.0014 | 0.0073 | <0.0001 |
| GH12 | 0.8226 | 0.0014 |  | 0.5886 | 0.0249 |
| GH97 | 0.5307 | 0.0073 | 0.5886 |  | 0.1265 |
| NCTC 13359 | 0.0304 | <0.0001 | 0.0249 | 0.1265 |  |

| *K. pneumoniae* | NCTC 9633 | NCTC 13438 | MGH 78578 | NCTC 13439 | NCTC 13368 |
| --- | --- | --- | --- | --- | --- |
| NCTC 9633 |  | 0.5299 | 0.6845 | 0.5958 | 0.7216 |
| NCTC 13438 | 0.5299 |  | 0.3760 | 0.8450 | 0.7307 |
| MGH 78578 | 0.6845 | 0.3760 |  | 0.4028 | 0.4617 |
| NCTC 13439 | 0.5958 | 0.8450 | 0.4028 |  | 0.9989 |
| NCTC 13368 | 0.7216 | 0.7307 | 0.4617 | 0.9989 |  |

| *A. baumannii* | UKA15 | UKA2 | AYE | ATCC 17978 | W1 |
| --- | --- | --- | --- | --- | --- |
| UKA15 |  | 0.3013 | 0.5676 | 0.6651 | 0.7876 |
| UKA2 | 0.3013 |  | 0.2261 | 0.2446 | 0.2038 |
| AYE | 0.5676 | 0.2261 |  | 0.7055 | 0.7848 |
| ATCC 17978 | 0.6651 | 0.2446 | 0.7055 |  | 0.6738 |
| W1 | 0.7876 | 0.2038 | 0.7848 | 0.6738 |  |

Supplementary Table 1 - Probability values for strain to strain variation in slow killing of *C. elegans*. Values highlighted in green indicate where there was a significant difference between two strains

Supplementary Table 2

|  | PAO1 | GH56 | GH12 | GH97 | NCTC 13359 |
| --- | --- | --- | --- | --- | --- |
| *gacA* |  |  |  |  |  |
| *Vfr* |  |  |  |  |  |
| *pchH* |  |  |  |  |  |
| *PA4005 (rsfS)* |  |  |  |  |  |
| *PA14_27700* |  |  |  |  |  |
| *pepP* |  |  |  |  |  |
| *hemK* |  |  |  |  |  |
| *lysC* |  |  |  |  |  |
| *vqsR* |  |  |  |  |  |
| *kinB* |  |  |  |  |  |
| *ptsP* |  |  |  |  |  |
| *lasR* |  |  |  |  |  |
| *PA0745* |  |  |  |  |  |
| *rhlR* |  |  |  |  |  |
| *PA2550* |  |  |  |  |  |
| *minD* |  |  |  |  |  |
| *PA1592* |  |  |  |  |  |
| *glnK* |  |  |  |  |  |
| *aruD* |  |  |  |  |  |
| *gshA* |  |  |  |  |  |
| *PA2015 (liuA)* |  |  |  |  |  |
| *cspB* |  |  |  |  |  |
| *prpC* |  |  |  |  |  |
| *gacS* |  |  |  |  |  |
| *lasI* |  |  |  |  |  |
| *pchI* |  |  |  |  |  |
| *aruG* |  |  |  |  |  |
| *fabF1* |  |  |  |  |  |
| *clpA* |  |  |  |  |  |
| *aruB* |  |  |  |  |  |
| *PA1766* |  |  |  |  |  |
| *PA1216* |  |  |  |  |  |
| *pqsE* |  |  |  |  |  |
| *kdpD* |  |  |  |  |  |
| *PA1767* |  |  |  |  |  |
| *prpB* |  |  |  |  |  |
| *Fha2* |  |  |  |  |  |
| *gshB* |  |  |  |  |  |
| *aruC* |  |  |  |  |  |
| *wbpL* |  |  |  |  |  |
| *pilA* |  |  |  |  |  |
| *pilF* |  |  |  |  |  |
| *ORF_1* |  |  |  |  |  |
| *plcS* |  |  |  |  |  |
| *toxA* |  |  |  |  |  |
| *dsbA* |  |  |  |  |  |
| *aefA* |  |  |  |  |  |
| *eda* |  |  |  |  |  |
| *proC* |  |  |  |  |  |
| *hcnC* |  |  |  |  |  |
| *rocS1* |  |  |  |  |  |
| *pvdF* |  |  |  |  |  |
| *pvdP* |  |  |  |  |  |

Supplementary Table 2 – Presence/absence of virulence factors in *P. aeruginosa* strains. Green indicates that the gene is present, amber indicates that the gene is present but there is a difference in gene length and red indicates that a gene is absent. All strains were compared to PAO1 where appropriate. Grey indicates that the gene may be present but it is probably a different serotype and therefore the sequence is different.
